# Supplementary material for: Deletion of TRPC6, an Autism Risk Gene, Induces Hyperexcitability in Cortical Neurons Derived from Human Pluripotent Stem Cells
Source: Mol Neurobiol. 2023 Aug 8;60(12):7297–308. doi: 10.1007/s12035-023-03527-0 (PMC10657791; doi:10.1007/s12035-023-03527-0)

# Supplementary Figure 1

a

5’CTGCCCACTCGGCTCCCCCGGGAGCGGGGCCCAGGCCAGTCGGGCGTTCCCGCCATGAGCCAGAGCCCGGCGTTCGGCCCCGGAGGGGCAGTTCTCCCCGG 3’
3’GACGGGTGAGCCGAGGGGGCCCTCGCCCCGGGTCCGGTCAGCCCGCAAGGGCGGTACTCGGTCTCGGGCCGCAAGCCCGGGGCCTCCCCGTCAAGAGGGGCC 5’
CAGCCCGCAAGGGCGGTACT (guide RNA)

b

5’CTGCCCACTCGGCTCCCCCGGGAGCGGGGCCCAGGCCAGTCGGGCGTTCCCGCCATGAGCCAGAGCCCGGCGTTCGGCCCCGGAGGGGCAGTTCTCCCCGG 3’ (Wild-type)
5’CTGCCCACTCGGCTCCCCCGGGAGC-----------------------------------------------------------------AGTTCTCCCCGG 3’ (TRPC6 KO, C21)
5’CTGCCCACTCGGCTCCCCCGGGAGCGGGGCCCAG-------------------------------------------------GAGGGGCAGTTCTCCCCGG 3’ (TRPC6 KO, C47)

c

TRPC6 KO (C21), 65 nucleotides deletion

TRPC6 KO (C47), 49 nucleotides deletion

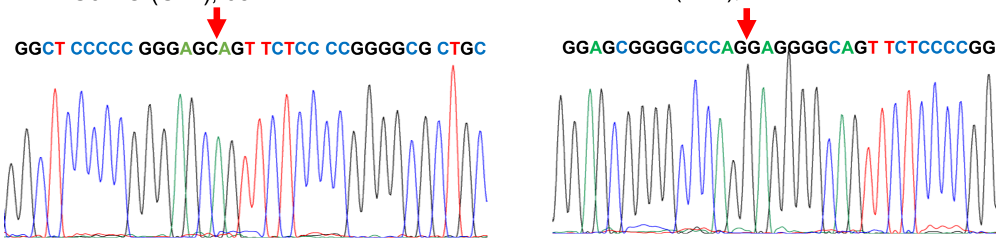


d

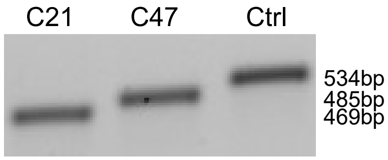

Supplementary Figure 2

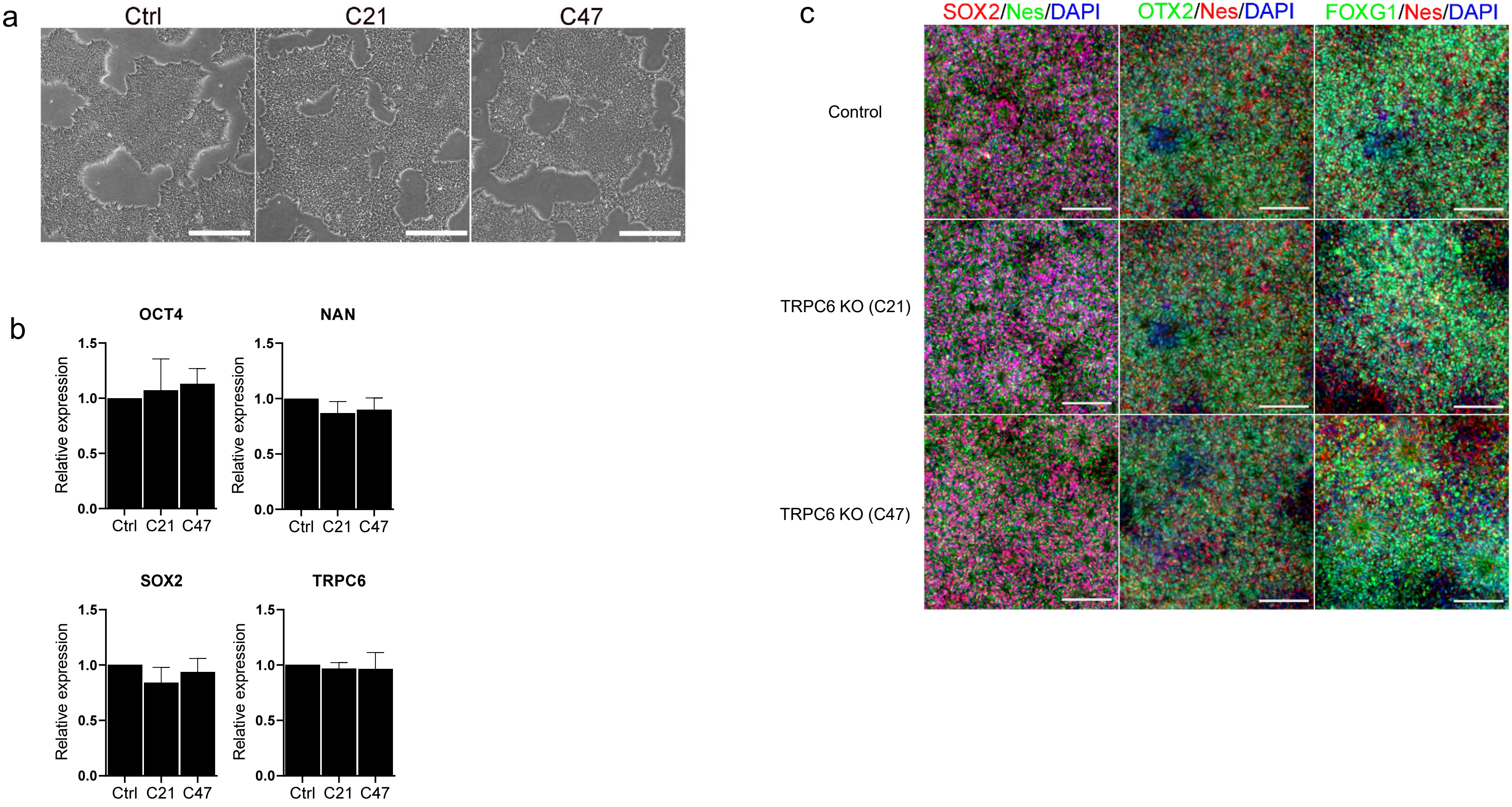

# Supplementary Figure 3

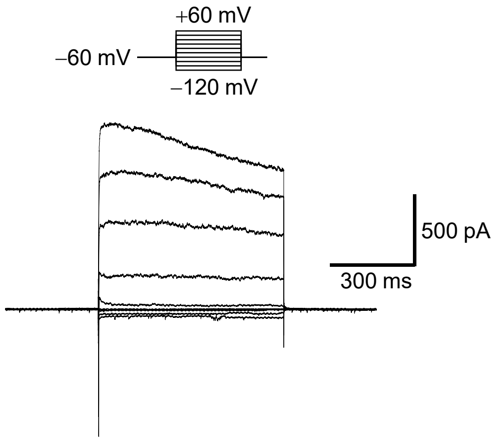

# Supplementary Figure 4

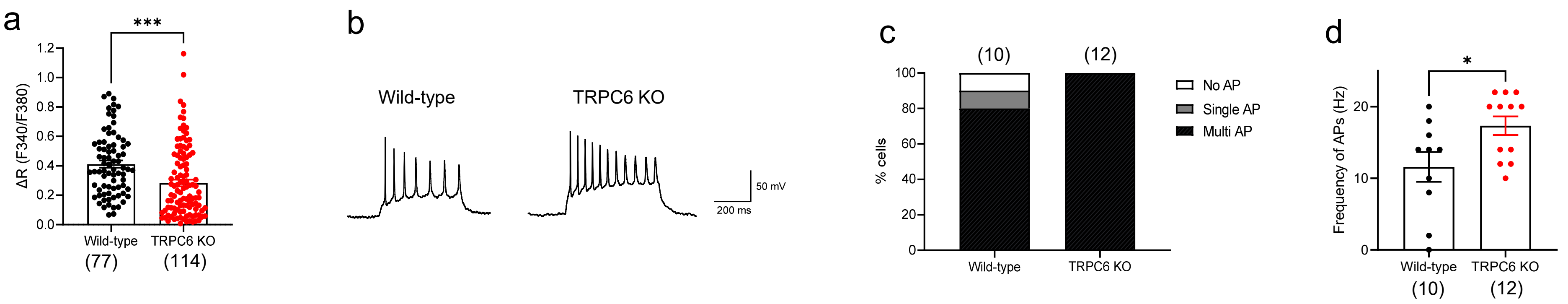

# Supplementary Figure 5

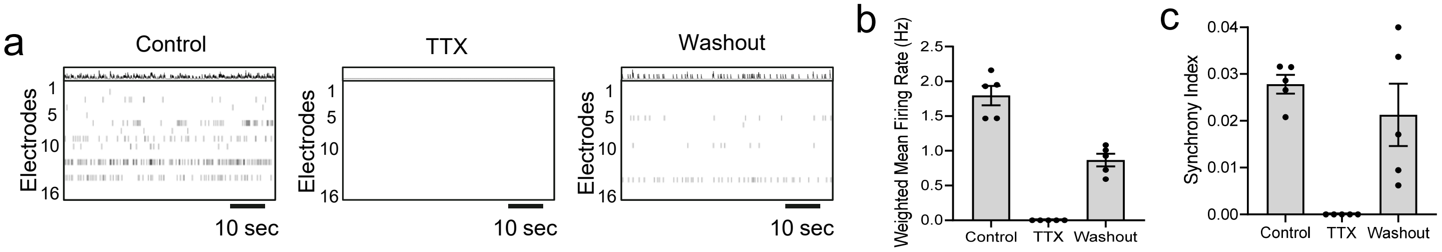

# Supplementary Figure 6

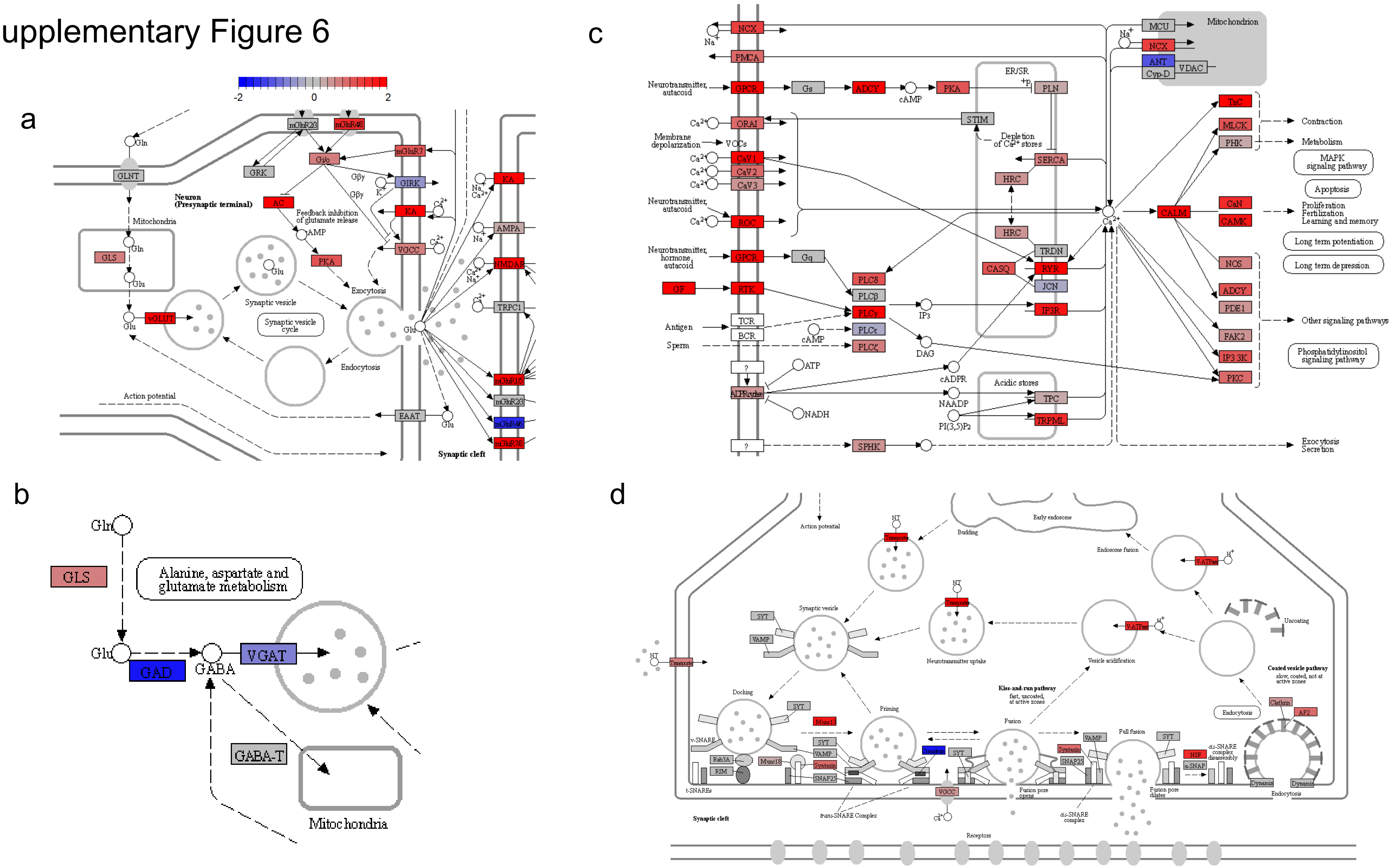

Supplement: Supplementary file 1 — Supplementary file1 (PDF 2761 KB) [file 12035_2023_3527_MOESM1_ESM.pdf]
