# Supplementary material for: Deletion of TRPC6, an Autism Risk Gene, Induces Hyperexcitability in Cortical Neurons Derived from Human Pluripotent Stem Cells
Source: Mol Neurobiol. 2023 Aug 8;60(12):7297–308. doi: 10.1007/s12035-023-03527-0 (PMC10657791; doi:10.1007/s12035-023-03527-0)
Supplement: Supplementary file 2 — Supplementary file2 (DOCX 39 KB) [file 12035_2023_3527_MOESM2_ESM.docx]

**Supplementary Figure legend**

**Supplementary Figure 1. TRPC6 KO in CRTD5 hPSC lines using CRISPR/Cas9.** (**a**) Genomic DNA sequence of TRPC6 intronic and exonic sequence spanning the start codon (green) and guide RNA targeting sequence. (**b,c**) Genomic DNA sequencing analysis of KO clones. PCR primers were designed to amplify the gRNA-targeted region. Sequence of KO clone 21 (65 bp deletion), clone 47 (49 bp deletion), and wild-type TRPC6. KO clones were identified by Sanger sequencing. (**d**) PCR products from the intact DNA sequence, 534 bp.

**Supplementary Figure 2.** (**a**) Bright field images of control (Ctrl) and KO clones (C21 and C47). (**b**) Expression of pluripotency markers; OCT4, NANOG, and SOX2. TRPC6 mRNA in KO clones. (**c**) Immunostaining of control NPCs and TRPC6 KO NPCs derived from two different hPSC clones (C21 and C47) with antibodies against SOX2, OTX2, FOXG1, and Nestin (Nes). Nuclei are stained with DAPI. Scale, 200 µm.

**Supplementary Figure 3.** Whole-cell currents in hPSC-derived cortical neurons differentiated for 6 weeks from NPCs were measured using whole-cell patch-clamp in a voltage-clamp mode. Ionic current is generated by a series of 20 mV voltage steps from −120 to +60 mV for 1 sec in a voltage-clamp mode. Na^+^ channels are rapidly inactivated, whereas K^+^ channels remain open upon membrane depolarization.

**Supplementary Figure 4.** (**a**) Reduced SOCE in different hPSC line (C47)-derived TRPC6 KO cortical neurons. Quantification of net calcium increase in hPSC-derived cortical neurons (C47 hPSC line) differentiated for 8 weeks. (**b**) Representative multiple AP of wild-type and different hPSC line (C47)-derived TRPC6 KO neurons differentiated for 6 weeks. (**c**) Distributions of AP generation of either no AP, single AP, or multiple/repetitive AP and (**d**) frequency of APs in wild-type and TRPC6 KO hPSC-derived cortical neurons (C47 hPSC line). Data in **a-c** are means ± SEM and number of cells tested are shown in parentheses from two independent differentiation and unpaired two-tailed *t*-test was used. *, *p* < 0.05. ***, *p* < 0.001.

**Supplementary Figure 5.** (**a**) Raster plots showing electrical activity of hPSC-derived cortical neurons at 8 weeks of differentiation in the presence of 1 µM TTX (5 min) and recovery after washout. Each row of spikes represents an electrode; 16 electrodes in a single well. Vertical red rectangles represent events of network bursts of electrical activity. Quantification of weighted mean firing rate (Hz) (**b**) and synchrony index (**c**). Data in **b,c** are means ± SEM (n = 5) from 2 independent differentiation.

**Supplementary Figure 6.** KEGG Pathway enrichment analysis using clusterProfiler and Pathview. KEGG view on (**a**) glutamatergic presynaptic neurons, (**b**) GABAergic presynaptic neurons, (**c**) calcium signaling pathway, and (**d**) synaptic vesicle cycle pathway. Colors in **a-d** correspond to log_2_ FC (fold changes) between control and TRPC6 KO hPSC-derived cortical neurons. Blue, downregulated; red, upregulated.

**Supplementary Table 1. Lists of up-regulated mRNA**

| **SYMBOL** | **GENENAME** | **Log_2_ (Fold Change)** | ***p* value** |
| --- | --- | --- | --- |
| GAP43 | growth associated protein 43 | 1.033870671 | 3.45E-05 |
| LRRC4C | leucine rich repeat containing 4C | 1.021258356 | 0.000326 |
| ADCYAP1 | adenylate cyclase activating polypeptide 1 | 2.230841897 | 0.000326 |
| COL26A1 | collagen type XXVI alpha 1 chain | 1.123485943 | 0.000326 |
| NCDN | neurochondrin | 0.968826012 | 0.000581186 |
| TNFRSF21 | TNF receptor superfamily member 21 | 0.900953424 | 0.001171634 |
| ASS1 | argininosuccinate synthase 1 | 1.582859115 | 0.001241375 |
| GRIK4 | glutamate ionotropic receptor kainate type subunit 4 | 1.312135195 | 0.001429505 |
| LAMB1 | laminin subunit beta 1 | 1.382019775 | 0.001429505 |
| SLC7A8 | solute carrier family 7 member 8 | 1.087926311 | 0.001489996 |
| CHST2 | carbohydrate sulfotransferase 2 | 1.090749641 | 0.001720594 |
| ASAH1 | N-acylsphingosine amidohydrolase 1 | 0.598837414 | 0.002287273 |
| PCDH11Y | protocadherin 11 Y-linked | 1.388443363 | 0.002353082 |
| ELMOD1 | ELMO domain containing 1 | 1.2147616 | 0.002713021 |
| AK5 | adenylate kinase 5 | 1.12378118 | 0.003148925 |
| PCDHA6 | protocadherin alpha 6 | 0.760612014 | 0.003323802 |
| KCNMA1 | potassium calcium-activated channel subfamily M alpha 1 | 1.166679192 | 0.003473133 |
| COL6A1 | collagen type VI alpha 1 chain | 1.212280702 | 0.003473133 |
| AHNAK2 | AHNAK nucleoprotein 2 | 1.193722292 | 0.003473133 |
| CAMK2B | calcium/calmodulin dependent protein kinase II beta | 1.044486882 | 0.003631229 |
| NCALD | neurocalcin delta | 0.849144596 | 0.003631229 |
| LINGO2 | leucine rich repeat and Ig domain containing 2 | 1.655670919 | 0.003631229 |
| DLC1 | DLC1 Rho GTPase activating protein | 1.385481508 | 0.003767912 |
| COL18A1 | collagen type XVIII alpha 1 chain | 1.551413203 | 0.004696488 |
| LNX1 | ligand of numb-protein X 1 | 1.379433033 | 0.004965924 |
| ARID5B | AT-rich interaction domain 5B | 0.86354827 | 0.005095606 |
| PITPNM1 | phosphatidylinositol transfer protein membrane associated 1 | 0.624915212 | 0.005095606 |
| PLXDC1 | plexin domain containing 1 | 1.151091703 | 0.005134186 |
| PHF24 | PHD finger protein 24 | 1.245463589 | 0.005134186 |
| PLAT | plasminogen activator, tissue type | 0.918797857 | 0.005134186 |
| FIBCD1 | fibrinogen C domain containing 1 | 1.610713511 | 0.005134186 |
| MAP6 | microtubule associated protein 6 | 0.736538166 | 0.005140611 |
| CDS1 | CDP-diacylglycerol synthase 1 | 0.873740904 | 0.005140611 |
| PPP1R14C | protein phosphatase 1 regulatory inhibitor subunit 14C | 1.421781611 | 0.005140611 |
| MAP7 | microtubule associated protein 7 | 0.961093572 | 0.005519659 |
| RET | ret proto-oncogene | 0.856126702 | 0.005519659 |
| GAS7 | growth arrest specific 7 | 0.922712783 | 0.005527751 |
| NIPAL3 | NIPA like domain containing 3 | 0.778216288 | 0.00553991 |
| SH3KBP1 | SH3 domain containing kinase binding protein 1 | 0.7153963 | 0.00553991 |
| COL6A2 | collagen type VI alpha 2 chain | 1.422223822 | 0.00553991 |
| DIRAS2 | DIRAS family GTPase 2 | 0.936577313 | 0.005576762 |
| RBMS1 | RNA binding motif single stranded interacting protein 1 | 1.931289521 | 0.005625998 |
| CEP126 | centrosomal protein 126 | 0.864363363 | 0.005764157 |
| ACHE | acetylcholinesterase (Cartwright blood group) | 1.372501103 | 0.005764157 |
| COL4A1 | collagen type IV alpha 1 chain | 2.036677238 | 0.006231656 |
| SRPX2 | sushi repeat containing protein X-linked 2 | 2.532876844 | 0.006231656 |
| ECM1 | extracellular matrix protein 1 | 1.16907177 | 0.006231656 |
| GFRA2 | GDNF family receptor alpha 2 | 1.233864617 | 0.006231656 |
| PTCHD4 | patched domain containing 4 | 1.287111062 | 0.006326898 |
| KIAA0513 | KIAA0513 | 0.608663452 | 0.006336731 |
| PRICKLE2 | prickle planar cell polarity protein 2 | 0.827567988 | 0.006336731 |
| CHN1 | chimerin 1 | 0.892357634 | 0.006504012 |
| PPP2R2C | protein phosphatase 2 regulatory subunit Bgamma | 1.046372749 | 0.006504012 |
| LAMA2 | laminin subunit alpha 2 | 1.567174033 | 0.00657376 |
| MGAT4A | alpha-1,3-mannosyl-glycoprotein 4-beta-N-acetylglucosaminyltransferase A | 1.076731692 | 0.00657376 |
| EFNA5 | ephrin A5 | 1.355782536 | 0.006673961 |
| GPC5 | glypican 5 | 1.709646516 | 0.00670641 |
| ZNF365 | zinc finger protein 365 | 0.898632112 | 0.00670641 |
| RBFOX1 | RNA binding fox-1 homolog 1 | 0.904401317 | 0.00670641 |
| BHLHE41 | basic helix-loop-helix family member e41 | 1.236629626 | 0.006908166 |
| SDC3 | syndecan 3 | 0.702181865 | 0.007076216 |
| CTHRC1 | collagen triple helix repeat containing 1 | 1.943171833 | 0.007240382 |
| RDH10 | retinol dehydrogenase 10 | 1.471949186 | 0.007321961 |
| ETS2 | ETS proto-oncogene 2, transcription factor | 0.959144493 | 0.007484174 |
| CFH | complement factor H | 2.215729348 | 0.008004306 |
| FAM43A | family with sequence similarity 43 member A | 1.264330984 | 0.008004306 |
| IL4R | interleukin 4 receptor | 2.373529981 | 0.008004306 |
| EBF2 | EBF transcription factor 2 | 2.128548629 | 0.008004306 |
| ADAMTS15 | ADAM metallopeptidase with thrombospondin type 1 motif 15 | 1.86566395 | 0.008239766 |
| ADAM23 | ADAM metallopeptidase domain 23 | 0.638434487 | 0.008352306 |
| BAIAP3 | BAI1 associated protein 3 | 1.309330698 | 0.008488892 |
| SLC6A17 | solute carrier family 6 member 17 | 1.084208228 | 0.008488892 |
| CORO2A | coronin 2A | 0.880670896 | 0.008488892 |
| FBLN2 | fibulin 2 | 1.330321597 | 0.008718913 |
| TPD52L1 | TPD52 like 1 | 1.597409043 | 0.008754024 |
| NKAIN1 | sodium/potassium transporting ATPase interacting 1 | 1.044269299 | 0.008754024 |
| COL4A2 | collagen type IV alpha 2 chain | 1.731225535 | 0.00886495 |
| NEFM | neurofilament medium chain | 1.194844112 | 0.00886495 |
| PLA2R1 | phospholipase A2 receptor 1 | 2.192774803 | 0.009161962 |
| DNAJC6 | DnaJ heat shock protein family (Hsp40) member C6 | 0.755273497 | 0.009161962 |
| CLSTN2 | calsyntenin 2 | 1.030702596 | 0.009404588 |
| KCNJ6 | potassium inwardly rectifying channel subfamily J member 6 | 1.210715178 | 0.009712176 |
| NT5E | 5'-nucleotidase ecto | 2.002010821 | 0.009712176 |
| ACSL4 | acyl-CoA synthetase long chain family member 4 | 0.598533709 | 0.010022411 |
| TGFBR2 | transforming growth factor beta receptor 2 | 1.887619199 | 0.010022411 |
| CNTNAP2 | contactin associated protein 2 | 0.695201097 | 0.010051295 |
| COL3A1 | collagen type III alpha 1 chain | 1.979252642 | 0.010199983 |
| HAS3 | hyaluronan synthase 3 | 1.031671458 | 0.010199983 |
| ADAMTS14 | ADAM metallopeptidase with thrombospondin type 1 motif 14 | 2.666286616 | 0.010321191 |
| HGF | hepatocyte growth factor | 2.341012863 | 0.010321191 |
| RAB3B | RAB3B, member RAS oncogene family | 1.327460659 | 0.01034288 |
| DCC | DCC netrin 1 receptor | 1.482004349 | 0.010917706 |
| KLHL1 | kelch like family member 1 | 2.003713977 | 0.011117551 |
| NFASC | neurofascin | 0.631689375 | 0.011268902 |
| RPRM | reprimo, TP53 dependent G2 arrest mediator homolog | 1.02634016 | 0.011399212 |
| SYP | synaptophysin | 0.670320756 | 0.011560165 |
| VWC2L | von Willebrand factor C domain containing 2 like | 1.11635146 | 0.011725817 |
| RALYL | RALY RNA binding protein like | 1.032483431 | 0.011731443 |
| KIAA1671 | KIAA1671 | 0.631798103 | 0.011849615 |
| STAMBPL1 | STAM binding protein like 1 | 0.937580005 | 0.012466689 |
| RNF152 | ring finger protein 152 | 0.647201827 | 0.012466689 |
| STAT6 | signal transducer and activator of transcription 6 | 0.651837645 | 0.012466689 |
| PAK6 | p21 (RAC1) activated kinase 6 | 0.766299945 | 0.012877029 |
| SYT4 | synaptotagmin 4 | 1.359018175 | 0.01339027 |
| GRIK1 | glutamate ionotropic receptor kainate type subunit 1 | 1.169440434 | 0.013410095 |
| SYT5 | synaptotagmin 5 | 0.738265212 | 0.013827102 |
| SAMD5 | sterile alpha motif domain containing 5 | 1.771251489 | 0.013859514 |
| PCSK1 | proprotein convertase subtilisin/kexin type 1 | 1.824829048 | 0.013859514 |
| LAMC1 | laminin subunit gamma 1 | 0.823659151 | 0.014009993 |
| ENDOD1 | endonuclease domain containing 1 | 0.587112607 | 0.014009993 |
| ARPP21 | cAMP regulated phosphoprotein 21 | 0.964717429 | 0.014009993 |
| EGR4 | early growth response 4 | 6.38623542 | 0.014009993 |
| COLEC12 | collectin subfamily member 12 | 1.854832541 | 0.014145281 |
| SLC8A2 | solute carrier family 8 member A2 | 0.694299004 | 0.014235868 |
| SCPEP1 | serine carboxypeptidase 1 | 0.961245711 | 0.01424604 |
| GABRB1 | gamma-aminobutyric acid type A receptor subunit beta1 | 1.24191784 | 0.01424604 |
| LINGO1 | leucine rich repeat and Ig domain containing 1 | 1.228025608 | 0.014335792 |
| CFAP221 | cilia and flagella associated protein 221 | 1.235248959 | 0.015125373 |
| PARM1 | prostate androgen-regulated mucin-like protein 1 | 1.537761716 | 0.015125373 |
| RHBDF1 | rhomboid 5 homolog 1 | 1.032485179 | 0.015392532 |
| SORCS1 | sortilin related VPS10 domain containing receptor 1 | 1.349062979 | 0.015392532 |
| FHDC1 | FH2 domain containing 1 | 1.052651837 | 0.015483114 |
| CACNB2 | calcium voltage-gated channel auxiliary subunit beta 2 | 0.643776049 | 0.015822095 |
| OLFML3 | olfactomedin like 3 | 2.515500772 | 0.015822095 |
| GDF15 | growth differentiation factor 15 | 2.260327274 | 0.015822095 |
| CLEC2B | C-type lectin domain family 2 member B | 2.617758955 | 0.015822095 |
| HRK | harakiri, BCL2 interacting protein | 1.865289993 | 0.015822095 |
| TRNP1 | TMF1 regulated nuclear protein 1 | 0.818573262 | 0.015822095 |
| TRIM38 | tripartite motif containing 38 | 2.049803169 | 0.016234756 |
| CBLN2 | cerebellin 2 precursor | 1.407949426 | 0.016442925 |
| KIAA0319 | KIAA0319 | 0.781120458 | 0.016533861 |
| APCDD1L | APC down-regulated 1 like | 2.358226872 | 0.016719285 |
| SYNGR3 | synaptogyrin 3 | 0.64356193 | 0.016759381 |
| PLEKHA6 | pleckstrin homology domain containing A6 | 0.784448632 | 0.0168601 |
| PGM2L1 | phosphoglucomutase 2 like 1 | 0.730972121 | 0.016884188 |
| NEFL | neurofilament light chain | 1.33851876 | 0.016884188 |
| TRIM22 | tripartite motif containing 22 | 0.860761262 | 0.016884188 |
| RYR2 | ryanodine receptor 2 | 1.298377761 | 0.016983925 |
| PDGFB | platelet derived growth factor subunit B | 1.039933306 | 0.016983925 |
| GABRB2 | gamma-aminobutyric acid type A receptor subunit beta2 | 1.001557424 | 0.016983925 |
| ORAI2 | ORAI calcium release-activated calcium modulator 2 | 0.647621525 | 0.016983925 |
| CDH6 | cadherin 6 | 1.659837679 | 0.016983925 |
| MLPH | melanophilin | 2.166317549 | 0.016983925 |
| KCNA1 | potassium voltage-gated channel subfamily A member 1 | 1.388571926 | 0.016983925 |
| INSYN2A | inhibitory synaptic factor 2A | 1.355858613 | 0.016983925 |
| STX1A | syntaxin 1A | 0.678930398 | 0.017173245 |
| HSPB7 | heat shock protein family B (small) member 7 | 3.092077051 | 0.017234636 |
| ARHGDIG | Rho GDP dissociation inhibitor gamma | 1.048064553 | 0.017822936 |
| COL13A1 | collagen type XIII alpha 1 chain | 2.275051784 | 0.018421634 |
| SLC9A7 | solute carrier family 9 member A7 | 0.709808986 | 0.018691934 |
| FBXO2 | F-box protein 2 | 0.836234405 | 0.018769808 |
| LYPD1 | LY6/PLAUR domain containing 1 | 1.04245264 | 0.019143103 |
| RPRML | reprimo like | 1.655769767 | 0.019176567 |
| SYBU | syntabulin | 0.630627315 | 0.019188623 |
| LRRTM1 | leucine rich repeat transmembrane neuronal 1 | 0.723979611 | 0.019405784 |
| RUNX1 | RUNX family transcription factor 1 | 2.852241608 | 0.019405784 |
| ADGRL4 | adhesion G protein-coupled receptor L4 | 2.605292793 | 0.019423738 |
| SERPINE2 | serpin family E member 2 | 0.769551087 | 0.019541067 |
| GRM1 | glutamate metabotropic receptor 1 | 1.261590767 | 0.019547703 |
| CYP46A1 | cytochrome P450 family 46 subfamily A member 1 | 0.755551103 | 0.019833942 |
| ADAMTS2 | ADAM metallopeptidase with thrombospondin type 1 motif 2 | 1.250555812 | 0.020015922 |
| SMCO4 | single-pass membrane protein with coiled-coil domains 4 | 0.990841762 | 0.020034427 |
| GALNT9 | polypeptide N-acetylgalactosaminyltransferase 9 | 1.472546502 | 0.020034427 |
| COL1A2 | collagen type I alpha 2 chain | 2.348737267 | 0.020536382 |
| TRPM2 | transient receptor potential cation channel subfamily M member 2 | 2.234358092 | 0.020597677 |
| CLEC2A | C-type lectin domain family 2 member A | 2.1076604 | 0.020597677 |
| PDGFC | platelet derived growth factor C | 1.1032754 | 0.020671853 |
| GCH1 | GTP cyclohydrolase 1 | 0.788838605 | 0.021160123 |
| ALK | ALK receptor tyrosine kinase | 1.825239624 | 0.021526041 |
| PDE1C | phosphodiesterase 1C | 1.199923722 | 0.021526041 |
| ADAM12 | ADAM metallopeptidase domain 12 | 2.552761124 | 0.021526041 |
| FAP | fibroblast activation protein alpha | 2.781982033 | 0.021938881 |
| JCAD | junctional cadherin 5 associated | 1.679096661 | 0.021988974 |
| SYT7 | synaptotagmin 7 | 0.79906489 | 0.022253652 |
| INSM2 | INSM transcriptional repressor 2 | 1.526166457 | 0.022319005 |
| DAB2 | DAB adaptor protein 2 | 2.256865238 | 0.022472501 |
| GABRA5 | gamma-aminobutyric acid type A receptor subunit alpha5 | 0.965954782 | 0.022564208 |
| CHRNA4 | cholinergic receptor nicotinic alpha 4 subunit | 0.67805953 | 0.022587892 |
| ITGA5 | integrin subunit alpha 5 | 2.119431171 | 0.023257841 |
| SLC35F3 | solute carrier family 35 member F3 | 1.549508988 | 0.023916999 |
| GRM7 | glutamate metabotropic receptor 7 | 1.221383716 | 0.023916999 |
| APOL6 | apolipoprotein L6 | 2.019640771 | 0.024216357 |
| LGALS3 | galectin 3 | 1.400185461 | 0.024216357 |
| ARHGAP44 | Rho GTPase activating protein 44 | 0.673279708 | 0.024266747 |
| COL21A1 | collagen type XXI alpha 1 chain | 1.901518748 | 0.024287522 |
| NRP1 | neuropilin 1 | 1.056500864 | 0.024462137 |
| SAMD3 | sterile alpha motif domain containing 3 | 1.833876104 | 0.02448602 |
| SIX4 | SIX homeobox 4 | 1.337095562 | 0.024531888 |
| TGFB1 | transforming growth factor beta 1 | 0.811771837 | 0.024615965 |
| ITGA11 | integrin subunit alpha 11 | 2.527979804 | 0.025549786 |
| CALB1 | calbindin 1 | 1.7907118 | 0.026109256 |
| SHC1 | SHC adaptor protein 1 | 0.796810452 | 0.026639935 |
| MFAP2 | microfibril associated protein 2 | 1.433574293 | 0.026709085 |
| PCDH15 | protocadherin related 15 | 0.81978303 | 0.026746196 |
| ALDH1A2 | aldehyde dehydrogenase 1 family member A2 | 1.858080474 | 0.026746196 |
| CLCN4 | chloride voltage-gated channel 4 | 0.662256303 | 0.026848723 |
| GLRB | glycine receptor beta | 0.753645723 | 0.026931519 |
| ND5 | NADH dehydrogenase subunit 5 | 0.625413093 | 0.027190491 |
| HS3ST4 | heparan sulfate-glucosamine 3-sulfotransferase 4 | 1.667263358 | 0.027190491 |
| CNIH3 | cornichon family AMPA receptor auxiliary protein 3 | 0.723806765 | 0.027190491 |
| MFAP4 | microfibril associated protein 4 | 1.84549241 | 0.027233473 |
| ARHGAP29 | Rho GTPase activating protein 29 | 1.224338878 | 0.027233473 |
| PDGFA | platelet derived growth factor subunit A | 0.706948421 | 0.028084594 |
| NDST4 | N-deacetylase and N-sulfotransferase 4 | 1.176891941 | 0.028263919 |
| DUSP23 | dual specificity phosphatase 23 | 0.752502201 | 0.02828744 |
| ZNF284 | zinc finger protein 284 | 0.758199735 | 0.028846405 |
| ANK1 | ankyrin 1 | 0.959005399 | 0.028846405 |
| SLITRK5 | SLIT and NTRK like family member 5 | 0.772912322 | 0.029201643 |
| ADGRF4 | adhesion G protein-coupled receptor F4 | 2.98307976 | 0.029334182 |
| NIPAL2 | NIPA like domain containing 2 | 1.498483129 | 0.029334182 |
| COL1A1 | collagen type I alpha 1 chain | 2.48582689 | 0.029334182 |
| ICA1 | islet cell autoantigen 1 | 0.701036251 | 0.029421413 |
| LRP1B | LDL receptor related protein 1B | 0.703072993 | 0.029441137 |
| ABCG4 | ATP binding cassette subfamily G member 4 | 0.781199376 | 0.029441137 |
| LDLRAP1 | low density lipoprotein receptor adaptor protein 1 | 1.265805627 | 0.029441137 |
| PEG3 | paternally expressed 3 | 0.728796957 | 0.029543076 |
| FAT3 | FAT atypical cadherin 3 | 0.689935134 | 0.029543076 |
| GPR176 | G protein-coupled receptor 176 | 0.991587556 | 0.029543076 |
| FXYD5 | FXYD domain containing ion transport regulator 5 | 3.749013591 | 0.029556668 |
| PLPP4 | phospholipid phosphatase 4 | 2.353867088 | 0.029601521 |
| NRIP1 | nuclear receptor interacting protein 1 | 0.587677915 | 0.029892817 |
| WNT4 | Wnt family member 4 | 0.868008976 | 0.029965262 |
| ANKRD45 | ankyrin repeat domain 45 | 1.421702987 | 0.029990689 |
| FOXS1 | forkhead box S1 | 1.626251292 | 0.030386893 |
| C2orf80 | chromosome 2 open reading frame 80 | 0.640616573 | 0.03047589 |
| VAT1L | vesicle amine transport 1 like | 1.101990919 | 0.03047589 |
| IL1RAP | interleukin 1 receptor accessory protein | 2.025953455 | 0.03047589 |
| S100A6 | S100 calcium binding protein A6 | 0.763627176 | 0.03047589 |
| EDNRA | endothelin receptor type A | 1.73041308 | 0.03047589 |
| ADGRA2 | adhesion G protein-coupled receptor A2 | 1.259771714 | 0.030527593 |
| NTN1 | netrin 1 | 1.840352914 | 0.031046265 |
| SYT16 | synaptotagmin 16 | 0.723301674 | 0.031583297 |
| BGN | biglycan | 2.19594847 | 0.03174653 |
| SYTL3 | synaptotagmin like 3 | 0.933406497 | 0.032284126 |
| S100A10 | S100 calcium binding protein A10 | 1.274551393 | 0.032284126 |
| PTPRE | protein tyrosine phosphatase receptor type E | 1.192385338 | 0.032284126 |
| GBX2 | gastrulation brain homeobox 2 | 2.522263666 | 0.032384769 |
| CHSY3 | chondroitin sulfate synthase 3 | 1.115430484 | 0.032622581 |
| RTL1 | retrotransposon Gag like 1 | 2.09870598 | 0.032761117 |
| SV2C | synaptic vesicle glycoprotein 2C | 1.75153399 | 0.032761117 |
| SCN1A | sodium voltage-gated channel alpha subunit 1 | 0.82472362 | 0.032761117 |
| TMEM200A | transmembrane protein 200A | 0.7999391 | 0.032961892 |
| MIR4435-2HG | MIR4435-2 host gene | 1.144987222 | 0.033161809 |
| GNG11 | G protein subunit gamma 11 | 0.77713011 | 0.033161809 |
| AAGAB | alpha and gamma adaptin binding protein | 0.707555653 | 0.033161809 |
| ELK3 | ETS transcription factor ELK3 | 1.518364954 | 0.033351652 |
| ABLIM1 | actin binding LIM protein 1 | 1.051407729 | 0.033503827 |
| CARD10 | caspase recruitment domain family member 10 | 1.125735461 | 0.034469071 |
| STING1 | stimulator of interferon response cGAMP interactor 1 | 1.736640985 | 0.034473226 |
| CYP1B1 | cytochrome P450 family 1 subfamily B member 1 | 2.124435803 | 0.034473226 |
| CACNA1A | calcium voltage-gated channel subunit alpha1 A | 0.844794655 | 0.034625744 |
| BNC2 | basonuclin 2 | 2.856039194 | 0.03463452 |
| PCDH17 | protocadherin 17 | 0.929433903 | 0.034863169 |
| RGS7 | regulator of G protein signaling 7 | 0.631569694 | 0.034958258 |
| CHODL | chondrolectin | 1.730584716 | 0.034997031 |
| MCHR1 | melanin concentrating hormone receptor 1 | 1.247528798 | 0.036005772 |
| C2orf15 | chromosome 2 open reading frame 15 | 1.170324794 | 0.036005772 |
| SGSH | N-sulfoglucosamine sulfohydrolase | 0.847356154 | 0.036044341 |
| RELN | reelin | 1.891149434 | 0.036279562 |
| CPZ | carboxypeptidase Z | 2.190654281 | 0.036385808 |
| DMTN | dematin actin binding protein | 0.836551569 | 0.036385808 |
| DGAT2 | diacylglycerol O-acyltransferase 2 | 1.147962084 | 0.036627558 |
| CDH20 | cadherin 20 | 1.108189233 | 0.036628779 |
| GPR143 | G protein-coupled receptor 143 | 1.501567195 | 0.037162717 |
| NAV2 | neuron navigator 2 | 1.086160894 | 0.037375628 |
| DOCK5 | dedicator of cytokinesis 5 | 1.353580323 | 0.037709836 |
| HRH1 | histamine receptor H1 | 2.216209808 | 0.037723856 |
| BATF3 | basic leucine zipper ATF-like transcription factor 3 | 1.164345066 | 0.037826672 |
| TLL2 | tolloid like 2 | 1.338178066 | 0.03847424 |
| PRRX1 | paired related homeobox 1 | 1.141683367 | 0.03847424 |
| PSEN2 | presenilin 2 | 0.781473264 | 0.03847424 |
| LUM | lumican | 1.97110951 | 0.038816504 |
| CAST | calpastatin | 0.63783971 | 0.038816504 |
| GMNC | geminin coiled-coil domain containing | 2.648798352 | 0.038816504 |
| SLC22A18 | solute carrier family 22 member 18 | 1.191498821 | 0.038870764 |
| STAC | SH3 and cysteine rich domain | 1.428496391 | 0.038901441 |
| OLFML2A | olfactomedin like 2A | 1.893774451 | 0.039025766 |
| FN1 | fibronectin 1 | 2.606437462 | 0.039139029 |
| TPD52 | tumor protein D52 | 1.229078861 | 0.039139289 |
| SLC35F2 | solute carrier family 35 member F2 | 1.711415333 | 0.039385342 |
| IRX2 | iroquois homeobox 2 | 1.339264418 | 0.039385342 |
| PHACTR2 | phosphatase and actin regulator 2 | 1.05441104 | 0.039385342 |
| COL16A1 | collagen type XVI alpha 1 chain | 1.015771511 | 0.039753517 |
| ADAMTS8 | ADAM metallopeptidase with thrombospondin type 1 motif 8 | 1.875900932 | 0.039753517 |
| SP100 | SP100 nuclear antigen | 1.820303054 | 0.039945527 |
| DNASE1L1 | deoxyribonuclease 1 like 1 | 0.587963654 | 0.040001081 |
| ADCY1 | adenylate cyclase 1 | 0.677300585 | 0.040001081 |
| TLL1 | tolloid like 1 | 1.473852151 | 0.040156556 |
| LOC283731 | uncharacterized LOC283731 | 1.396156498 | 0.040209822 |
| PARVG | parvin gamma | 1.033650307 | 0.040209822 |
| ZBTB7C | zinc finger and BTB domain containing 7C | 0.877857174 | 0.040209822 |
| SLAMF8 | SLAM family member 8 | 1.052319321 | 0.040423526 |
| TNFRSF10C | TNF receptor superfamily member 10c | 1.915139206 | 0.040504358 |
| OPN5 | opsin 5 | 1.336765504 | 0.040504358 |
| PALMD | palmdelphin | 1.434274196 | 0.040504358 |
| CD248 | CD248 molecule | 1.826588818 | 0.040504358 |
| CSRP1 | cysteine and glycine rich protein 1 | 0.79796412 | 0.040504358 |
| TMEM255A | transmembrane protein 255A | 1.565063143 | 0.040504358 |
| SAMD11 | sterile alpha motif domain containing 11 | 1.397066492 | 0.040534651 |
| ST6GALNAC5 | ST6 N-acetylgalactosaminide alpha-2,6-sialyltransferase 5 | 0.843477463 | 0.040578509 |
| TMEM130 | transmembrane protein 130 | 0.751663962 | 0.040760077 |
| PRTG | protogenin | 0.979910715 | 0.040940074 |
| FMNL2 | formin like 2 | 0.79648365 | 0.041098837 |
| KIRREL1 | kirre like nephrin family adhesion molecule 1 | 0.622503057 | 0.041511778 |
| POSTN | periostin | 4.201745735 | 0.042086398 |
| LIF | LIF interleukin 6 family cytokine | 1.690898199 | 0.042148089 |
| ITPR1 | inositol 1,4,5-trisphosphate receptor type 1 | 1.01718264 | 0.042229307 |
| LTBR | lymphotoxin beta receptor | 2.475243811 | 0.042285895 |
| STPG1 | sperm tail PG-rich repeat containing 1 | 0.722014273 | 0.042366678 |
| SLC38A4-AS1 | SLC38A4 antisense RNA 1 | 1.316729039 | 0.043040765 |
| PLAU | plasminogen activator, urokinase | 2.766971123 | 0.043098215 |
| CCND1 | cyclin D1 | 1.695275054 | 0.04324847 |
| POU3F1 | POU class 3 homeobox 1 | 0.971299866 | 0.043535485 |
| ACE | angiotensin I converting enzyme | 1.273945784 | 0.043535485 |
| PAPPA | pappalysin 1 | 2.597816924 | 0.043535485 |
| SLC4A11 | solute carrier family 4 member 11 | 2.584586799 | 0.043535485 |
| HOOK1 | hook microtubule tethering protein 1 | 0.911672243 | 0.043639049 |
| ARHGAP26 | Rho GTPase activating protein 26 | 0.739158416 | 0.043639049 |
| SOD2 | superoxide dismutase 2 | 0.717468156 | 0.043772202 |
| SLC22A15 | solute carrier family 22 member 15 | 1.085708159 | 0.043772202 |
| OXTR | oxytocin receptor | 1.277838749 | 0.043918823 |
| PLCG2 | phospholipase C gamma 2 | 2.656507321 | 0.044416873 |
| HIVEP3 | HIVEP zinc finger 3 | 0.681970915 | 0.044416873 |
| TAC1 | tachykinin precursor 1 | 3.093234969 | 0.044416873 |
| TLE4 | TLE family member 4, transcriptional corepressor | 1.456810899 | 0.044416873 |
| PTPRD-DT | PTPRD divergent transcript | 2.234938773 | 0.044416873 |
| VXN | vexin | 1.695635954 | 0.044416873 |
| DNAJC12 | DnaJ heat shock protein family (Hsp40) member C12 | 0.960897814 | 0.044416873 |
| RAB29 | RAB29, member RAS oncogene family | 0.765560524 | 0.044588066 |
| HTR5A | 5-hydroxytryptamine receptor 5A | 1.289260061 | 0.044675717 |
| FAM174B | family with sequence similarity 174 member B | 0.764610651 | 0.044765283 |
| MARCHF3 | membrane associated ring-CH-type finger 3 | 0.931563736 | 0.044901868 |
| CMTM7 | CKLF like MARVEL transmembrane domain containing 7 | 2.808013753 | 0.044901868 |
| TPM1 | tropomyosin 1 | 0.721735397 | 0.045402212 |
| RDH10-AS1 | RDH10 antisense RNA 1 | 1.88244019 | 0.046158208 |
| GABBR2 | gamma-aminobutyric acid type B receptor subunit 2 | 0.798289271 | 0.046447538 |
| N4BP3 | NEDD4 binding protein 3 | 0.747238401 | 0.046600775 |
| EPHA2 | EPH receptor A2 | 1.226162324 | 0.046600775 |
| SFMBT2 | Scm like with four mbt domains 2 | 0.738896476 | 0.046834624 |
| GDPD5 | glycerophosphodiester phosphodiesterase domain containing 5 | 0.612298426 | 0.046909346 |
| CCDC157 | coiled-coil domain containing 157 | 0.734041893 | 0.047012872 |
| PHLDA3 | pleckstrin homology like domain family A member 3 | 0.590944783 | 0.047016119 |
| HMCN2 | hemicentin 2 | 1.95903734 | 0.047038775 |
| EDIL3 | EGF like repeats and discoidin domains 3 | 1.596083377 | 0.047421077 |
| LOC442132 | golgin A6 family-like 1 pseudogene | 4.420017302 | 0.047421077 |
| FMNL1 | formin like 1 | 0.637623203 | 0.047505863 |
| MYOF | myoferlin | 1.842699908 | 0.048185579 |
| TESC | tescalcin | 1.327884989 | 0.048185579 |
| COL5A2 | collagen type V alpha 2 chain | 1.691587347 | 0.048329613 |
| FAM89A | family with sequence similarity 89 member A | 1.436513631 | 0.049291912 |
| F12 | coagulation factor XII | 1.049560092 | 0.049291912 |
| SEC24D | SEC24 homolog D, COPII coat complex component | 0.604619929 | 0.049291912 |
| C4B | complement C4B (Chido blood group) | 3.531869186 | 0.049291912 |
| ASPHD2 | aspartate beta-hydroxylase domain containing 2 | 0.620680575 | 0.049291912 |
| ANXA11 | annexin A11 | 0.633134101 | 0.049291912 |
| CCDC198 | coiled-coil domain containing 198 | 1.912893434 | 0.049291912 |
| OPRM1 | opioid receptor mu 1 | 2.877371396 | 0.049303958 |
| TRH | thyrotropin releasing hormone | 1.338004589 | 0.049303958 |
| IL13RA2 | interleukin 13 receptor subunit alpha 2 | 0.882054195 | 0.049303958 |
| AFF2 | ALF transcription elongation factor 2 | 0.620897713 | 0.049303958 |
| CALHM5 | calcium homeostasis modulator family member 5 | 1.993610514 | 0.04976429 |
| ITGA3 | integrin subunit alpha 3 | 0.587530295 | 0.049834602 |

**Supplementary Table 1. Lists of down-regulated mRNA**

| **SYMBOL** | **GENENAME** | **Log_2_ (Fold Change)** | ***p* value** |
| --- | --- | --- | --- |
| PUS7L | pseudouridine synthase 7 like | -8.799062026 | 1.44E-05 |
| MIR4458HG | MIR4458 host gene | -8.066763045 | 3.43E-05 |
| IRAK4 | interleukin 1 receptor associated kinase 4 | -9.930139472 | 0.000249484 |
| RXRA | retinoid X receptor alpha | -1.2390219 | 0.000581186 |
| SLAIN1 | SLAIN motif family member 1 | -0.994632015 | 0.000641089 |
| ZNF513 | zinc finger protein 513 | -0.789733285 | 0.001429505 |
| POU2F1 | POU class 2 homeobox 1 | -0.610369027 | 0.001429505 |
| DAPK1 | death associated protein kinase 1 | -0.667902217 | 0.002353082 |
| PHF21B | PHD finger protein 21B | -0.80009508 | 0.002713021 |
| ARRDC4 | arrestin domain containing 4 | -0.903363831 | 0.003017932 |
| ZNF677 | zinc finger protein 677 | -1.092710527 | 0.003148925 |
| IFI44 | interferon induced protein 44 | -1.098438172 | 0.003473133 |
| BTG1 | BTG anti-proliferation factor 1 | -0.844015199 | 0.003631229 |
| ZNF454 | zinc finger protein 454 | -1.782993324 | 0.003631229 |
| ZNF772 | zinc finger protein 772 | -0.639509857 | 0.003680423 |
| IL33 | interleukin 33 | -0.817982812 | 0.003937526 |
| GLCCI1 | glucocorticoid induced 1 | -0.592490323 | 0.004696488 |
| GAS2L3 | growth arrest specific 2 like 3 | -1.223598017 | 0.004696488 |
| SOX2 | SRY-box transcription factor 2 | -0.769920776 | 0.004862101 |
| PBX4 | PBX homeobox 4 | -1.048718348 | 0.0050267 |
| LRRC1 | leucine rich repeat containing 1 | -0.85742544 | 0.0050267 |
| RGS12 | regulator of G protein signaling 12 | -0.873991488 | 0.005134186 |
| LOC100287846 | uncharacterized LOC100287846 | -5.433242561 | 0.005134186 |
| RFTN2 | raftlin family member 2 | -1.069156026 | 0.00553991 |
| FGFR3 | fibroblast growth factor receptor 3 | -1.158180132 | 0.005764157 |
| LRCH2 | leucine rich repeats and calponin homology domain containing 2 | -0.615131011 | 0.005764157 |
| LINC01563 | long intergenic non-protein coding RNA 1563 | -1.318692933 | 0.005871904 |
| S1PR1 | sphingosine-1-phosphate receptor 1 | -0.928172357 | 0.005961584 |
| FGF13 | fibroblast growth factor 13 | -1.124770423 | 0.005961584 |
| PTGFR | prostaglandin F receptor | -1.727935554 | 0.005961584 |
| RAB8B | RAB8B, member RAS oncogene family | -0.646347038 | 0.006231656 |
| NDRG2 | NDRG family member 2 | -0.732927141 | 0.006231656 |
| TMEM123 | transmembrane protein 123 | -1.276173793 | 0.006231656 |
| ALDH1L1 | aldehyde dehydrogenase 1 family member L1 | -1.57908947 | 0.006336731 |
| CXCR4 | C-X-C motif chemokine receptor 4 | -1.936376996 | 0.00657376 |
| GCNT2 | glucosaminyl (N-acetyl) transferase 2 (I blood group) | -0.810344193 | 0.00670641 |
| MTSS1 | MTSS I-BAR domain containing 1 | -0.771430771 | 0.006926964 |
| COBL | cordon-bleu WH2 repeat protein | -1.139561178 | 0.006965526 |
| ZBTB16 | zinc finger and BTB domain containing 16 | -1.956158427 | 0.006998119 |
| CKB | creatine kinase B | -0.60591126 | 0.007240382 |
| ASCL1 | achaete-scute family bHLH transcription factor 1 | -1.035332358 | 0.007321961 |
| LPAR2 | lysophosphatidic acid receptor 2 | -0.72994369 | 0.008004306 |
| BRME1 | break repair meiotic recombinase recruitment factor 1 | -0.613561172 | 0.008004306 |
| CHRDL1 | chordin like 1 | -1.026962763 | 0.008309871 |
| GLDC | glycine decarboxylase | -0.613840242 | 0.008488892 |
| SMPD5 | sphingomyelin phosphodiesterase 5 (pseudogene) | -1.283879336 | 0.008818206 |
| MIRLET7BHG | MIRLET7B host gene | -1.191667518 | 0.009330916 |
| HAS1 | hyaluronan synthase 1 | -3.959221565 | 0.010321191 |
| ZNF704 | zinc finger protein 704 | -0.745941627 | 0.011117551 |
| H3-3B | H3.3 histone B | -0.629728952 | 0.011117551 |
| GRHL1 | grainyhead like transcription factor 1 | -0.825318346 | 0.011147237 |
| EEA1 | early endosome antigen 1 | -0.598975014 | 0.01147504 |
| PDZRN3 | PDZ domain containing ring finger 3 | -2.043494932 | 0.011577014 |
| LOC646903 | uncharacterized LOC646903 | -0.940325885 | 0.012317552 |
| TNFAIP8L3 | TNF alpha induced protein 8 like 3 | -2.218020205 | 0.012912948 |
| CST3 | cystatin C | -0.629628168 | 0.014382379 |
| CHD7 | chromodomain helicase DNA binding protein 7 | -0.799774515 | 0.014831317 |
| ATOSA | atos homolog A | -0.816505115 | 0.015822095 |
| SATB1 | SATB homeobox 1 | -0.704914686 | 0.016719285 |
| LINC02060 | long intergenic non-protein coding RNA 2060 | -1.018947911 | 0.016797031 |
| PDE9A | phosphodiesterase 9A | -0.722690536 | 0.0168601 |
| NR3C2 | nuclear receptor subfamily 3 group C member 2 | -2.703806215 | 0.016983925 |
| RAPGEF3 | Rap guanine nucleotide exchange factor 3 | -1.474458785 | 0.016983925 |
| TSC22D3 | TSC22 domain family member 3 | -0.745410443 | 0.016983925 |
| GPX3 | glutathione peroxidase 3 | -1.483118352 | 0.017234636 |
| SILC1 | sciatic injury induced lincRNA upregulator of SOX11 | -1.170951871 | 0.018769808 |
| PIP5K1B | phosphatidylinositol-4-phosphate 5-kinase type 1 beta | -1.417289783 | 0.020034427 |
| INF2 | inverted formin 2 | -0.659121561 | 0.020166568 |
| SALL1 | spalt like transcription factor 1 | -0.631946984 | 0.020960696 |
| ARHGEF28 | Rho guanine nucleotide exchange factor 28 | -1.367486054 | 0.021536108 |
| TBC1D4 | TBC1 domain family member 4 | -0.730223669 | 0.021938881 |
| IRAK3 | interleukin 1 receptor associated kinase 3 | -1.453564412 | 0.022945547 |
| SLFN11 | schlafen family member 11 | -1.982404557 | 0.024216357 |
| CWC25 | CWC25 spliceosome associated protein homolog | -6.756087226 | 0.024266747 |
| LINC00461 | long intergenic non-protein coding RNA 461 | -1.11247148 | 0.024341139 |
| NPAS3 | neuronal PAS domain protein 3 | -1.138046083 | 0.024462137 |
| POU4F1 | POU class 4 homeobox 1 | -2.587908613 | 0.025078308 |
| ID4 | inhibitor of DNA binding 4 | -0.665491252 | 0.025172461 |
| GRM6 | glutamate metabotropic receptor 6 | -2.273775543 | 0.025185853 |
| PELI2 | pellino E3 ubiquitin protein ligase family member 2 | -0.648268172 | 0.025859403 |
| SLC17A1 | solute carrier family 17 member 1 | -2.371580528 | 0.027233473 |
| VEPH1 | ventricular zone expressed PH domain containing 1 | -1.121289846 | 0.027786543 |
| ARX | aristaless related homeobox | -1.075658135 | 0.02888281 |
| LOX | lysyl oxidase | -1.130858558 | 0.02888281 |
| PRSS8 | serine protease 8 | -2.281280205 | 0.030080151 |
| PRKCA | protein kinase C alpha | -0.830744942 | 0.03047589 |
| ADGRV1 | adhesion G protein-coupled receptor V1 | -0.608378725 | 0.031446999 |
| EGFR | epidermal growth factor receptor | -1.070771102 | 0.031446999 |
| KCNT2 | potassium sodium-activated channel subfamily T member 2 | -0.715231212 | 0.032101108 |
| FREM2 | FRAS1 related extracellular matrix 2 | -1.069210114 | 0.032384769 |
| ENDOU | endonuclease, poly(U) specific | -1.22063003 | 0.032384769 |
| PLIN4 | perilipin 4 | -0.957512371 | 0.032811043 |
| PCDHB8 | protocadherin beta 8 | -0.840901775 | 0.032961892 |
| RND3 | Rho family GTPase 3 | -0.922208977 | 0.03323275 |
| SCRT2 | scratch family transcriptional repressor 2 | -0.816663014 | 0.033351652 |
| KCNA3 | potassium voltage-gated channel subfamily A member 3 | -0.853169785 | 0.034625744 |
| PRKX | protein kinase cAMP-dependent X-linked catalytic subunit | -0.617173246 | 0.03463452 |
| TCIM | transcriptional and immune response regulator | -0.682548626 | 0.034997031 |
| KLF15 | KLF transcription factor 15 | -0.743125162 | 0.036005772 |
| VIP | vasoactive intestinal peptide | -2.579595102 | 0.036058133 |
| RNASET2 | ribonuclease T2 | -0.678620696 | 0.036385808 |
| POU3F4 | POU class 3 homeobox 4 | -1.289728649 | 0.036406877 |
| PLS3 | plastin 3 | -1.890013274 | 0.037395195 |
| RASSF2 | Ras association domain family member 2 | -0.980519956 | 0.03847424 |
| SORCS3 | sortilin related VPS10 domain containing receptor 3 | -0.88970944 | 0.038901441 |
| TEX22 | testis expressed 22 | -0.81527171 | 0.039753517 |
| ZNF439 | zinc finger protein 439 | -0.812384853 | 0.040760077 |
| CDCA7 | cell division cycle associated 7 | -0.627670818 | 0.040760077 |
| ACVR1C | activin A receptor type 1C | -1.351079112 | 0.040760077 |
| CRACD | capping protein inhibiting regulator of actin dynamics | -0.783546667 | 0.040760077 |
| TCF15 | transcription factor 15 | -1.978812607 | 0.04218197 |
| DSCAML1 | DS cell adhesion molecule like 1 | -0.800967983 | 0.042506394 |
| PLIN5 | perilipin 5 | -0.895982277 | 0.043071803 |
| PAN2 | poly(A) specific ribonuclease subunit PAN2 | -1.650267988 | 0.043098215 |
| TMEM161B-DT | TMEM161B divergent transcript | -0.898851124 | 0.043258431 |
| FAM110B | family with sequence similarity 110 member B | -0.828203402 | 0.043418947 |
| EEF1D | eukaryotic translation elongation factor 1 delta | -2.426822364 | 0.04350382 |
| PRR18 | proline rich 18 | -0.840164345 | 0.043639049 |
| VCAM1 | vascular cell adhesion molecule 1 | -0.663820856 | 0.044416873 |
| RIPOR2 | RHO family interacting cell polarization regulator 2 | -0.999058999 | 0.044675717 |
| ZNF536 | zinc finger protein 536 | -1.649589759 | 0.045402212 |
| CD9 | CD9 molecule | -0.747250119 | 0.045459149 |
| ST8SIA5 | ST8 alpha-N-acetyl-neuraminide alpha-2,8-sialyltransferase 5 | -1.500477453 | 0.045697382 |
| C11orf96 | chromosome 11 open reading frame 96 | -0.721959652 | 0.046527884 |
| CPLX2 | complexin 2 | -0.709160456 | 0.047409822 |
| NUTM2G | NUT family member 2G | -0.801425748 | 0.047421077 |
| BMS1P14 | BMS1 pseudogene 14 | -1.09189051 | 0.047505863 |
| CXCL14 | C-X-C motif chemokine ligand 14 | -1.192869478 | 0.048185579 |
| LINC01111 | long intergenic non-protein coding RNA 1111 | -1.840449981 | 0.048671769 |
| PDZRN4 | PDZ domain containing ring finger 4 | -1.905039336 | 0.048973254 |
| MIR1539 | microRNA 1539 | -3.383285363 | 0.048973254 |
| NCAN | neurocan | -0.631228129 | 0.049303958 |

**Supplementary Table 2.**

| **Antibody name** | **species** | **company** | **Dilution** |
| --- | --- | --- | --- |
| TRPC6 | Rabbit | Abcam (ab228771) | 1:1000 |
| SOX2 | Rabbit | Invtirogen (MA1-014) | 1:200 |
| FOXG1 | Rabbit | Abcam (ab18259) | 1:200 |
| OTX2 | Goat | R&D (AF1979) | 1:300 |
| Nestin | Mouse | Invitrogen (MA1-110) | 1:100 |
| MAP2 | Chicken | Abcam (ab5392) | 1:500 |
| MAP2 | Mouse | Invitrogen (13-1500) | 1:500 |
| Beta-tubulin III | Mouse | Chemicon (MAB1637) | 1:300 |
| TBR2 | Rabbit | Cell signaling (66325) | 1:300 |
| CTIP2 | Rabbit | Cell signaling (12120 | 1:200 |
| BRN2 | Rabbit | Cell signaling | 1:200 |
| SATB2 | Rabbit | Invitrogen (PA5-83092) | 1:200 |
| VGLUT1 | Rabbit | Invitrogen (PA5-85764) | 1:200 |
| Synaptophysin | Rabbit | Abcam (ab32127) | 1:200 |
| GFAP | Chicken | Abcam (ab4674) | 1:400 |
| Alexa Flour 488 Donkey anti-Goat IgG | Donkey | Invitrogen (Cat# A-11055) | 1:1000 |
| Alexa Flour 488 Donkey anti-Mouse IgG | Donkey | Invitrogen (Cat#A-21202) | 1:1000 |
| Alexa Flour 488 Donkey anti-Rabbit IgG | Donkey | Invitrogen (Cat#A-21206) | 1:1000 |
| Alexa Flour 555 Donkey anti-Mouse IgG | Donkey | Invitrogen (Cat# A-31570) | 1:1000 |
| Alexa Flour 555 Donkey anti-Rabbit IgG | Donkey | Invitrogen (Cat# A-31572) |  |
| Flour 647 Donkey Anti-Rabbit IgG | Donkey | Invitrogen (Cat# A-31573) | 1:1000 |
| Anti-Rabbit-HRP secondary antibody | Goat | Thermo Fisher Scientific ( Cat # 31460) | 1:10,000 |

**PCR primers list**

| Gene name | Sequence | Amlicon length (bp) |
| --- | --- | --- |
| OCT3/4 | For: GACAGGGGGAGGGGAGGAGCTAGG  Rev: CTTCCCTCCAACCAGTTGCCCCAAAC | 119 |
| Nanog | For: CATGAGTGTGGATCCAGCTTG  Rev: CCTGAATAAGCAGATCCATGG | 192 |
| SOX2 | For: GGGAAATGGGAGGGGTGCAAAAGAGG  Rev: TTGCGTGAGTGTGGATGGGATTGGTG | 151 |
| TRPC6 | For: TTACTGGAGACCTGCCTTAGA  Rev: TTTCTGCTGACTCCGTGTG | 129 |
| TRPC6 | For: TGTTGACATAGTAACTCTTCAGCTCCGTCTCCCTTGC  Rev: AGGGGTAGTAGCCGTAGCAAGGCAGC | 534 |
